# Does influenza vaccination contribute to the prevention of cardiovascular events? An umbrella review

**DOI:** 10.14745/ccdr.v51i09a02

**Published:** 2025-10-09

**Authors:** Fazia Tadount, Nadine Sicard, Winnie Siu, Pamela Doyon-Plourde, Angela Sinilaité

**Affiliations:** 1 Public Health Agency of Canada, Ottawa, ON; 2 Département de microbiologie, infectiologie et immunologie, Université de Montréal, Montréal, QC; 3 School of Epidemiology and Public Health, University of Ottawa, Ottawa, ON

**Keywords:** influenza vaccine, cardiovascular events, vaccine effectiveness, myocardial infraction, stroke

## Abstract

**Background:**

There is a growing body of evidence on the potential benefit of influenza vaccination against the occurrence of cardiovascular (CV) events.

**Objective:**

This umbrella review of systematic reviews and meta-analyses (SRMAs) aims to summarize the available evidence on the risk of CV events in adults after receipt of influenza vaccine.

**Methods:**

Four electronic databases were searched (CINAHL, PubMed, SYSVAC and Cochrane Library) for SRMAs published in English or French, between January 1, 2000, and January 14, 2025. Eligible SRMAs included those with a quantitative synthesis of data examining the association between influenza vaccination and the risk of CV events in adults. Data from the included SRMAs were extracted using predefined variables. The quality of each SRMA was assessed by two independent reviewers using the AMSTAR 2 tool.

**Results:**

The review included 25 SRMAs published between 2012 and 2024. Overall, 15 SRMAs were deemed to be of moderate or high quality and were further considered in the evidence synthesis. The most frequently evaluated clinical outcomes were myocardial infarction (MI), all-cause and CV mortality, and major adverse cardiovascular events (MACE). In vaccinated individuals at high-risk for CV events, the risk of CV death was significantly reduced by 23% to 47%, MACE by 26% to 37%, MI by 29% to 34%, and stroke by 13% to 19% compared to unvaccinated individuals.

**Conclusion:**

High-quality evidence from the existing literature supports influenza vaccination as an effective preventive measure for reducing CV disease burden. Highlighting this benefit to patients could increase vaccine uptake and improve both influenza and CV outcomes, especially where coverage remains suboptimal.

## Introduction

Cardiovascular disease (CVD) is the leading cause of mortality worldwide ((1)). In 2021, deaths attributable to ischemic heart disease (IHD) and stroke accounted for 23% (~16 million) of deaths globally ((1)). Excess mortality from CVD during influenza epidemics was first recognized early in the 20^th^ century ((2)). Studies have since shown clinically significant association between respiratory infections, especially influenza and CVD ((3–8)). The risk of cardiovascular (CV) events, such as heart failure (HF), myocardial infraction (MI) and stroke, is several times higher after the onset of respiratory infection than in the absence of infection and increases in proportion to the severity of infection ((2–6)).

Despite vaccine availability, seasonal influenza causes significant morbidity and mortality ((9)). Part of its morbidity burden is for CV events, including MI, HF, and stroke, especially among individuals with pre-existing cardiac disorders, such as chronic HF or cardiomyopathy ((10)). Globally, it is estimated that 3%–5% of IHD deaths can be attributed to influenza, corresponding to 200,000–400,000 IHD deaths, annually ((11)). Studies have found that influenza infection can cause direct cardiac changes, and the hosts’ response to influenza virus infection can increase circulation of inflammatory mediators and activate immune cells that can induce damage in the cardiovascular system ((8)).

Seasonal influenza vaccination is an effective means to protect against severe influenza disease and its complications ((12)). Furthermore, evidence on the cardioprotective effects of influenza vaccines is mounting ((8,13)). In the last decade, many randomized controlled trials (RCTs) and observational studies were conducted to explore this potential association. In Canada, the National Advisory Committee on Immunization (NACI) identifies individuals at high-risk of influenza-related complications or hospitalizations, including those with chronic health conditions, such as cardiac or pulmonary disorders, as a population for whom annual seasonal influenza vaccination is particularly important ((14)). However, seasonal influenza vaccine coverage is suboptimal, including in high-risk populations ((15)). Similar recommendations were made in other countries, such as the United Kingdom, the United States, and Australia ((16–18)).

Several systematic reviews and meta-analyses (SRMAs) assessing the secondary protection of influenza vaccines against CV events have been published ((13)). Therefore, the objective was to conduct an evidence review to provide a comprehensive summary of published SRMAs that assessed the effect of seasonal influenza vaccination on CV events.

## Methods

This review was conducted according to a pre-established protocol and following guidance from the Systematic Reviews on Vaccines (SYSVAC) expert panel on the use of existing systematic reviews to develop evidence-based vaccination recommendations ((19)).

### Search strategy and study identification

An *“a priori”* search strategy was developed to identify relevant studies on PubMed, CINAHL, Cochrane Library and the SYSVAC registry. The detailed search strategy can be found in **Appendix**, **Supplemental** **A**. Initially, we searched for studies published between January 1, 2000, and March 27, 2024, in English or French languages. The search was updated on January 14, 2025, to incorporate the latest available evidence. Following the electronic database searches, identified records were uploaded into the DistillerSR platform for the screening process. One reviewer conducted the title and abstract screening, then the full-text screening to assess studies eligibility. To be included in the review, each study had to be an SRMA; systematic reviews with only a narrative summary and no meta-analysis were excluded. Furthermore, the Population, Intervention, Comparison, and Outcome(s) (PICO) component of each SRMA, and relevance of the research question(s) were assessed. Relevant SRMAs were eligible if each of the following PICO definitions was met, as defined in each SRMA:

• Population (P): Adults, with or without CVD

• Intervention (I): Seasonal influenza vaccine (any formulation, dose or type)

• Comparison (C): No seasonal influenza vaccine or placebo

• Outcomes (O): Incidence or occurrence of CV events

### Data extraction

An electronic data extraction form was developed for this review. The data extraction was first conducted by one reviewer and further validated and/or corrected by a second reviewer. Overall, abstracted data were general review characteristics (author, date of publication, search dates, objective and PICO elements), and a summary of main findings (i.e., participant characteristics, effect measures with a 95% confidence interval (CI), and the Grading of Recommendations Assessment, Development, and Evaluation (GRADE) for the overall quality of evidence), if available ((20)).

### Methodological assessment

The quality of each SRMA was assessed using A MeaSurement Tool to Assess Systematic Reviews (AMSTAR 2), a tool specifically designed to appraise systematic reviews and meta-analyses of randomized and non-randomized studies of healthcare interventions ((21)). In line with recommendations, the critical domains for the AMSTAR 2 tool were classified as items 2, 4, 7, 9, 11, 13 and 15 (**Table S1**) ((21)). For the present review, the AMSTAR 2 tool was further adapted so that any item with a “no” response was considered to be critical flaw, while items with a “partial yes” response were not considered critical flaws. The overall score derived using the AMSTAR 2 tool was used to rate the quality of each included SRMA as high (no critical flaws), moderate (one critical flaw), low (two to three critical flaws) or critically low (over three critical flaws) ((21)). This assessment was conducted by two independent reviewers, and conflicts were resolved through discussion and consensus.

### Data synthesis

The characteristics and main findings of eligible SRMAs were narratively summarized. Following SYSVAC guidelines for developing recommendations based on existing systematic reviews, only SRMAs of moderate or high quality were included in the detailed summary of findings ((19)). The PICO items for each SRMA were compared to appraise the heterogeneity between selected SRMAs. A matrix was created to present overlapping studies across the SRMAs. Findings for four main CV events were synthesized: CV mortality, major adverse CV events (MACE), MI and stroke. Effect measures and 95% CIs for these outcomes were presented in a forest plot, to provide a visual overview of the evidence. To account for potential heterogeneity due to the design of primary studies (i.e., RCT, or observational studies, or both), stratified results were presented by study design, when possible. Finally, results were reported separately for populations with and without underlying CVD to better appraise the effect of influenza vaccination in high-risk populations.

## Results

**Figure 1:**
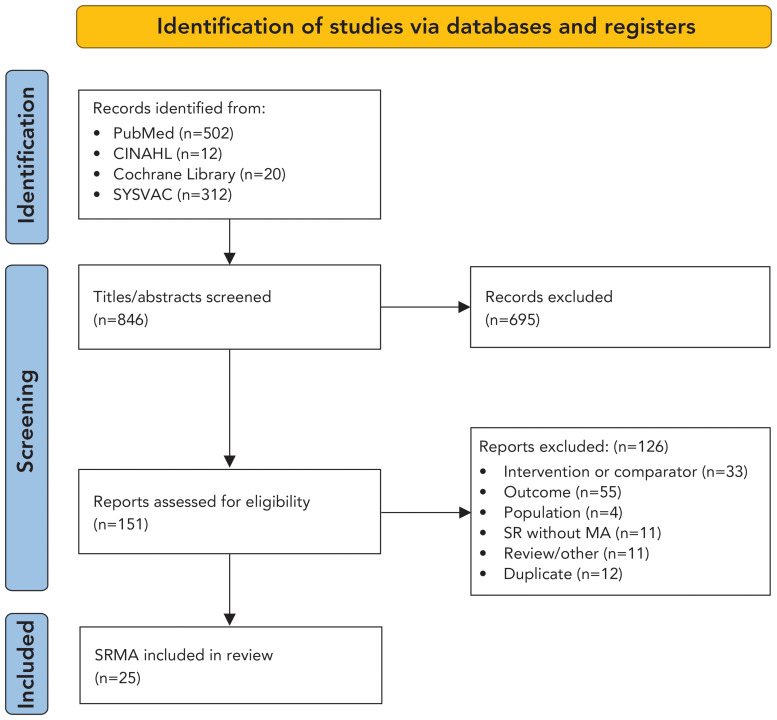
PRISMA diagram for study selection Abbreviations: MA, meta-analyse; PRISMA, Preferred Reporting Items for Systematic reviews and Meta-Analyses; SR, systematic review; SRMA, systematic review and meta-analyse; SYSVAC, Systematic Reviews on Vaccines

Overall, 846 citations were identified and screened at the title and abstract level. A total of 151 studies were assessed for eligibility and screened at full-text level, and 25 SRMAs were finally included in the umbrella review (**Figure 1**) ((22–46)).

### Studies description

**Figure 2:**
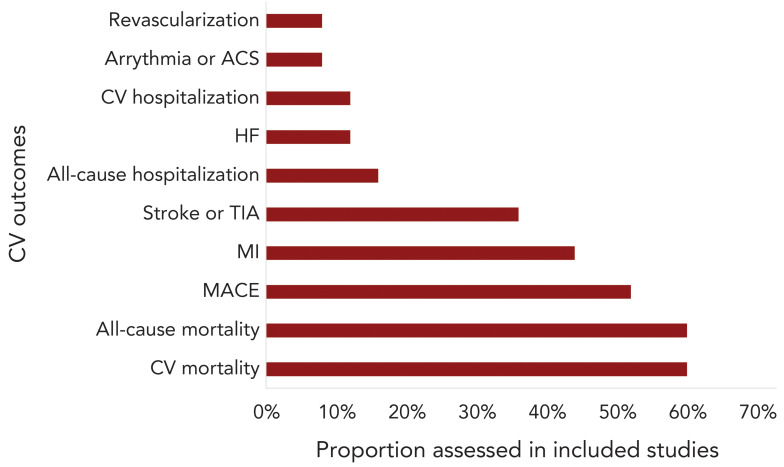
Proportion of assessed cardiovascular outcomes in identified systematic reviews and meta-analyses Abbreviations: ACS, acute coronary syndrome; CV, cardiovascular; HF, heart failure; MACE, major adverse cardiac events; MI, myocardial infarction; TIA, transient ischemic attack

Included SRMAs were published between 2012 and 2024 and included 5 to 22 individual studies in the quantitative synthesis (**Table 1**). Overall, nine (36%) of the studies were SRMAs of RCT ((22,24,27,28,35,37–39,43)), 10 (40%) were SRMAs of both RCT and observational studies ((26,29,31,33,34,36,42,44–46), and six (24%) included only observational studies (23,25,30,32,40,41). The populations of interest in all SRMAs were adults aged 18 years and older, although most SRMAs (72%) focused on participants with diagnosed CVD or those at higher risk of CV events, as defined in each SRMA (Table 1) ((22–25,28,29,31,32,34–40,42–44). In contrast, 28% of the SRMAs included a broader population definition, encompassing adults with or without CVD, and older adults ((26,27,30,33,41,45,46)). Furthermore, the eligible SRMAs assessed several CV outcomes, with MI, all-cause and/or CV mortality, and MACE being the most frequently evaluated outcomes (**Figure 2**).

**Table 1:** Characteristics of included systematic reviews and meta-analyses

Author, Year	Study design	PICO	Participant characteristics	Detailed outcome(s) definition	AMSTAR 2^a^
**SRMA of RCT**					
Liu *et al.* 2024	N=5 (RCT) Time covered: Until September 2024	P: Adult patients with IHD I: Influenza vaccinated people C: Unvaccinated people O: MACE or other clinical events	5,659 patients with IHD (2,838 vaccinated, 2,821 controls) Median age: 57–66 years 67.8% males Median follow-up: 12 months	MACE or other clinical events (including cardiovascular death, all-cause mortality, MI, hospitalization for HF, and re-vascularization)	High
Omidi *et al.* 2023	N=5 (RCT) Time covered: Until August 1, 2023	P: Patients with a diagnosis of CVD I: Influenza vaccine C: Placebo O: CV events	9,059 patients (4,529 vaccinated, 4,530 controls) Mean age: 61.3 years Mean follow-up: 9 months	MACE Included the following: MI, stroke, and/or CV death	Low
Barbetta *et al.* 2023	N=5 (RCT) Time covered: Until September 2021	P: Patients with coronary artery disease I: Influenza vaccine C: Placebo or no vaccine O: Reported at least one of the specified CV outcomes	4,187 patients (2,098 vaccinated, 2,089 controls) Intervention group: Mean age: 54.9–65 years 61%–81.4% males Control group: Mean age of 54.5–67 years 52%–82.1% males	Primary outcomes: MACE: CV death, non-fatal MI, non-fatal stroke All cause mortality CV mortality Secondary outcomes: Hospitalization for HF, stroke or TIA, revascularization, ACS	Moderate
Modin *et al.* 2023	N=6 (RCT) Time covered: Until December 2022	P: Patients with high CV risk (ischaemic heart disease and/or HF) I: Influenza vaccine C: Placebo O: Incidence of CV outcomes assessed as efficacy outcomes	9,340 patients (4,670 vaccinated, 4,670 controls) Mean age: 54.5–67 years Follow-up: 9.8–36 months	Primary endpoints: Composite of CV death, acute coronary syndrome, stent thrombosis or coronary revascularization, stroke or HF hospitalization Secondary endpoints: CV death, all-cause death	Moderate
Behrouzi *et al.* 2022	N=6 (RCT) Time covered: 2000–2021	P: Patients with cardiac history I: Influenza vaccine C: Placebo and no treatment O: Major adverse CV events	9,001 patients (4,510 vaccinated, 4,491 controls) 42.5% females Mean age: 65.5 years Cardiac history: 52.3% Mean follow-up: 9 months	Primary outcomes: Composite of MACE (CV death or hospitalization for MI, unstable angina, stroke, heart failure, or urgent coronary revascularization) within 12 months of follow-up Secondary outcomes: CV mortality within 12 months of follow-up	Critically low
Diaz-Arocutipa *et al.* 2022	N=5 (RCT) Time covered: Until September 2021	P: Patients with coronary artery disease I: Influenza vaccine C: Placebo or standard care O: MACE, all-cause mortality, CV mortality, and MI	4,175 patients (2,110 vaccinated, 2,065 controls) 75% males Mean age: 54.5–67 years Follow-up: 6–12 months Comorbidities: hypertension (55%), previous MI (23%), and diabetes (22%)	Primary outcomes: MACE Secondary outcomes: All-cause mortality, CV mortality, MI	Moderate
Maniar *et al.* 2022	N=8 (RCT) Time covered: Until May 2022	P: Patients hospitalized for acute MI or HF I: Influenza vaccination within a specified timeframe after hospitalization for MI or HF C: No influenza vaccination, placebo, or delayed vaccination O: Reduction in MACE and CV mortality	14,420 patients Follow-up: 6–36 months	MACE, CV mortality, all-cause mortality, MI	Critically low
Clar *et al.* 2015	N=8 (RCT) Time covered: Until February 2015	P: Patients 18 years and older who may or may not have had a history of CVD I: Influenza vaccination C: Control treatment O: CV death or non-fatal CV events	12,029 patients (1,682 with known CVD and 10,347 from general population or elderly people) Follow-up: 42 days–1 year	Primary outcomes: Patients without previous CVD: first-time MI, first-time unstable angina, death from CV causes Patients with previous CVD: MI, Unstable angina, death from CV causes Secondary outcomes: Composite clinical outcomes	Moderate
Udell *et al.* 2013	N=6 (RCT) Time covered: Until August 2013	P: Patients with high CV risk I: Influenza vaccination C: Placebo or standard of care O: CV events (efficacy or safety events)	6,735 patients 51.3% females Mean age: 67 years Cardiac history: 36.2% Mean follow-up time: 7.9 months	MACE, CV mortality, all-cause mortality, individual nonfatal CV events (MI, stroke, HF, hospitalization for unstable angina or cardiac ischemia, and urgent coronary revascularization)	Moderate
**SRMA of RCT and observational studies**					
Liu *et al.* 2024	N=6 (RCT) N=37 (Obs.) Time covered: Until September 2023	P: Adults (18+ years) from the general population or with established CVD I: Influenza vaccine C: Placebo or no vaccine O: All-cause or CV mortality, all-cause or CVD hospitalization	RCT: 12,662 participants Mean age, 62 years; 45% women; 8,797 (69%) with preexisting CVD Follow-up: 6–12 months Observational: 6,311,703 participants Mean age, 49 years; 50% women; 1,189,955 (19%) with pre-existing CVD	All-cause or CV mortality, all-cause or CVD hospitalization and CVD was defined as including any diagnoses relating to MI, HF, or stroke	High
Zahhar *et al.* 2024	Until December 2022 N=3 (RCT) N=23 (Obs.)	P: Patients >18 years I: Influenza vaccine C: No influenza vaccine O: Risk of stroke occurrence/ hospitalization	6,196,668 patients total 42% of studies included patients ≥65 years	Incidence/hospitalization due to stroke (any stroke, ischemic stroke, hemorrhagic stroke) and mortality	Moderate
Liu *et al.* 2022	N=1 (RCT) N=6 (Obs.) Time covered: Until October 2021	P: Adults (>18 years) I: Influenza vaccine C: No influenza vaccine or received vaccine beyond the period of efficacy O: Risk of arrhythmia	RCT: 2,532 patients Mean age: 59.85 years 80.51% males Mean/median follow-up: 1 year Observational: 3,167,445 patients Age range: 18–73.3 years 55.9%–85.29% males Mean/median follow-up: 9 months–3.7 years	Arrhythmia: including AF, atrial flutter, ventricular fibrillation, ventricular flutter, cardiac arrest	Moderate
Zangiabadian *et al.* 2020	N=6 (RCT) N=11 (Obs.) Time covered: January 2000–November 2019	P: Patients aged 18+ years I: Influenza vaccine C: No influenza vaccine O: CV events	Total: 180,043 cases and 276,898 control 47% of studies included patients ≥65 years RCT: 3,677 cases, 3,681 controls Age range: 18+ years Cohort: 78,522 cases, 127,833 controls Age range: 31+ years Case-control: 97,844 cases, 145,384 controls Age range: 40+ years	Occurrence of CV events (CV death, non-fatal MI, non-fatal stroke, hospitalization for HF, coronary ischemic events, HF, vascular death)	Low
Gupta *et al.* 2023	N=6 (RCT) N=9 (Obs.) Time covered: 2000–2021	P: Patients with and without CVD I: Influenza vaccination C: No influenza vaccination O: CV outcomes	745,001 patients Mean age: 70.11 (vaccinated) and 64.55 (unvaccinated) years Mean follow-up time: 6 months–2 years 50% females (vaccinated); 41% females (unvaccinated)	All-cause mortality, CV death, stroke, MI, hospitalization for HF	Critically low
Jaiswal *et al.* 2022	N=5 (RCT) N=13 (Obs.) Time covered: Until April 2022	P: Patients with established CVD or at high CV risk I: Influenza vaccine C: No influenza vaccine or placebo O: All-cause mortality, MACE, HF, MI, CV mortality, stroke	22,532,165 patients total 217,072 with high CV risk or established CVD (111,073 vaccinated, 105,999 unvaccinated) Mean age: 68 years Mean follow-up: 1.5 years	Primary outcomes: All-cause mortality, MACE Secondary outcomes: HF, MI, CV mortality, stroke	Low
Yedlapati *et al.* 2021	N=4 (RCT) N=12 (Obs.) Time covered: Until January 2020	P: Patients with CVD (atherosclerotic CVD or HF) I: Influenza vaccine C: Placebo O: Mortality and CV outcomes	237,058 patients total (RCT: 1,667 patients, observational: 235,391 patients) Mean age: 69.2 ± 7.01 years 36.6% females Median follow-up: 19.5 months	All-cause mortality, CV mortality, MACE, HF, MI	Low
Cheng *et al.* 2020	N=6 (RCT) N=69 (Obs.) Time covered: Until November 2018	P: Adults I: Influenza vaccine C: Placebo O: CV and respiratory disease outcomes and all-cause mortality	4,419,467 patients total Follow-up: 4 months–9 years	CVD (including stroke, MACE, MI, HF, ischemic heart disease, transient ischemic attack, acute coronary syndrome, cardiac arrest, CV mortality, atrial fibrillation) and all-cause mortality	Low
Tsivgoulis *et al.* 2018	N=5 (RCT-all included influenza) N=6 (Obs.) Time covered: Until March 2017	P: Adult patients at risk of cerebrovascular ischemia I: Influenza vaccination C: No influenza vaccination or different types of vaccination O: Ischemic stroke and other CV outcomes	431,937 patients total Mean age range: 59.9 + 10.3 years and older 19.9%–59.7% vaccinated 38.9%–72.5% males Follow-up time range: 6 months–2 years	Primary outcomes: Cerebrovascular ischemia, specifically acute ischemic stroke Secondary outcomes: Myocardial ischemic events, CV deaths	High
Loomba *et al.* 2012	N=3 (RCT) N=2 (Obs.) Time covered: 1998–2011	P: Patients with cardiovascular disease or at risk of CV events I: Influenza vaccine C: No influenza vaccine O: CV morbidity and mortality	292,383 patients total (169,203 vaccinated and 123,481 unvaccinated) Mean age: 58–77 years 42.6–73.9% males	MI, all-cause mortality, and MACE	Critically low
**SRMA of observational studies**					
Tavabe *et al.* 2023	N=14 (Obs.) Time covered: 1980–July 2021	P: Elderly I: Influenza vaccine C: No influenza vaccine O: Stroke and hospitalization occurrence	3,198,646 patients Mean follow-up: 30 months	Stroke occurrence or hospitalization due to stroke	Moderate
Gupta *et al.* 2022	N=7 (Obs.) Time covered: Until October 2021	P: Adult patients with heart failure I: Influenza vaccine C: No influenza vaccine O: All-cause mortality, CV-related mortality, all-cause hospitalization, CV-related hospitalization, non-fatal stroke, and non-fatal MI	247,842 patients Mean age: 68–77 years Male to female ratio close to 50% in most studies	All-cause mortality and hospitalization, CV mortality and hospitalization, non-fatal stroke, non-fatal MI within 12 months of receiving the influenza vaccine	Moderate
Rodrigues *et al.* 2020	N=6 (Obs.) Time covered: Until December 2018	P: Adult patients diagnosed with heart failure and/or if they had a reported abnormal/reduced ejection fraction (<50%) I: Influenza vaccination C: No influenza vaccination O: All-cause mortality, HF mortality, CV mortality, all-cause hospitalizations, CV hospitalization rates, HF-related hospitalization rates, hospitalization length and ventricular arrhythmias	179,158 patients Mean age: 62–75 years Follow-up: 3 months–8 years	Primary outcome: All-cause mortality Secondary outcomes: HF mortality, CV mortality, all-cause hospitalizations, CV hospitalization rates, HF-related hospitalization rates, hospitalization length and ventricular arrhythmias	High
Caldeira *et al.* 2019	N=2 (SCCS) Time covered: Until September 2019	P: Adult (18+ years) patients with a first recorded AMI in the study period and recorded influenza vaccination I: Influenza vaccination C: No influenza vaccination O: incidence rate of AMI	32,676 patients Median age: 72.3–77 years	Incident rate ratio of MI within first month (1–28 days) of influenza vaccination	Low
Lee *et al.* 2017	N=11 (Obs.) Time covered: Until November 2016	P: Individuals (18+ years) at risk of stroke I: Influenza vaccine C: No influenza vaccine O: Risk of stroke (any, first, recurrent)	593,513 patients 45% of studies included participants ≥60 years	Risk of stroke (any, first, recurrent)	Moderate
Barnes *et al.* 2015	N=7 (case-control) Time covered: Until June 2014	P: Adult patients with AMI I: Influenza vaccine C: Patients without AMI, including those who did and did not receive the influenza vaccine O: Fatal or non-fatal AMI, including first or subsequent episode(s)	17,695 cases with AMI (9,428 vaccinated) and 65,343 controls without AMI (33,819 vaccinated) Mean age: ≥40 years	Risk of AMI (first, recurrent). AMI was defined as a constellation of clinical features, including ischemic symptoms, biochemical and/or electrical evidence of myocardial ischemia, evidence of critical artery stenosis on coronary angiography or autopsy evidence of MI	Moderate

Abbreviations: ACS, acute coronary syndrome; AF, atrial fibrillation; AMI, acute myocardial infarction; AMSTAR, A MeaSurement Tool to Assess Systematic Reviews; CV, cardiovascular; CVD, cardiovascular disease; HF, heart failure; IHD, ischemic heart disease; MACE, major adverse cardiovascular events; MI, myocardial infarction; Obs., observational; PICO, population, intervention, comparison, outcome(s); RCT, randomized controlled trials; SCCS, self-controlled case series; SRMA, systematic review and meta-analyses; TIA, transient ischemic attack

^a^ The AMSTAR 2 tool was adapted for the present review, so that any item with a “no” response was classified as a critical flaw, while items with a “partial yes” response were not considered critical flaws

### Quality assessment and primary studies overlap

The quality assessment of each SRMA was performed using AMSTAR 2. This tool was adapted so that any item with a “no” response was classified as a critical flaw, while items with a “partial yes” response were not considered critical flaws. Overall, four SRMAs were deemed of “critically low” quality, six were of “low” quality, and a total of 15 SRMAs (60%) were deemed to be of “moderate” or “high” quality. Consequently, only the 15 SRMAs of “moderate” or “high” quality were included in the detailed summary of findings synthesis ((22,23,27–29,32–35,38,40–43,45)). The main items in which most SRMAs scored poorly were: not including a full list of excluded studies (item 7); the absence of a satisfactory technique for assessing the risk of bias in included individual studies (item 9); the use of appropriate methods in meta-analyses for statistical combination of results (item 11); and not accounting for the risk of bias in primary studies when discussing/interpreting the results (item 13) (**Table S1**).

Finally, the overlap between primary studies included in each SRMA was assessed further, and only two SRMA presented a 100% overlap between their primary studies (**Table S2**).

### Summary of findings

**Figure 3:**
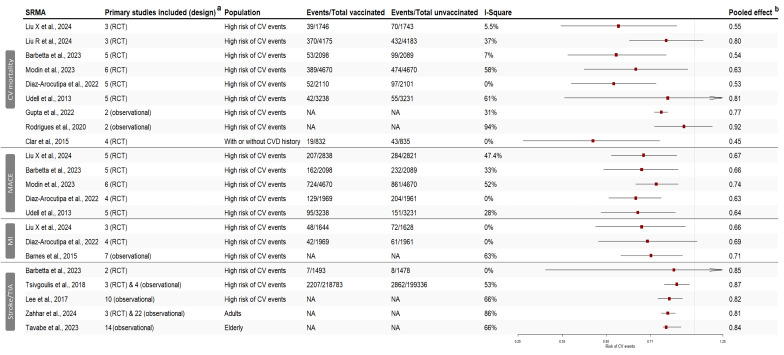
Forest plot showing the pooled effect measures from systematic reviews and meta-analyses for the association between influenza vaccination and cardiovascular events^a,b^ Abbreviations: CV, cardiovascular; MACE, major adverse cardiovascular events; MI, myocardial infarction; NA, not available; RCT, randomized controlled trials; TIA, transient ischemic attack ^a^ Primary studies included in the quantitative analysis ^b^ Odds ratios (ORs), hazard ratios (HRs) and risk ratios (RRs) are plotted on the same graphic. Since cardiovascular events were rare (<10%), all measures tend to be equivalent. This graphic is solely a representation of the effects; no further analysis was conducted

The umbrella review resulted in the following findings:

• **Cardiovascular mortality:** A total of nine out of 15 (67%) SRMAs assessed CV-related mortality, eight were in patients with underlying CVD or at higher risk of CV events, ((22,28,29,34,35,38,40,43)), while one consisted of adults with or without CVD history (28). Overall, six SRMAs (67%) showed a significant reduction in CV mortality following influenza vaccination (**Figure 3**) ((22,27–29,35,38)). In adults with a higher risk of CV events, the risk of death due to a CV event was reduced by 23% (95% CI: 19%–27%) to 47% (95% CI: 26%–62%) in vaccinated individuals compared to those who were not vaccinated. The heterogeneity of these findings was low to moderate, ranging between 0% and 58%. Similarly, pooled data from four RCTs that included adults with or without CVD history showed a 55% (95% CI: 24%–74%) risk reduction in CV mortality, with no heterogeneity (I^2^: 0%). Conversely, results were not significant in three SRMAs, with moderate to critical heterogeneity (I^2^: 37%–94%) (Figure 3) ((34,40,43)).

• **Major adverse cardiac events:** MACE is a composite outcome endpoint that generally included: CV death, all-cause mortality, acute coronary syndrome (ACS), MI, hospitalization for a CV event, revascularization, stroke, and HF. Overall, five (33%) SRMAs assessed the effect of influenza vaccination on MACE outcomes in participants with high-risk of CV events. All were SRMAs of RCT, and their overall findings were consistently showing a 26% (95% CI: 12%–51%) to 37% (95% CI: 23%–49%) significant reduction in the risk of MACE in vaccinated individuals. The heterogeneity of these results was low to moderate (I^2^: 0%–47%) (Figure 3) ((22,28,35,38,43)).

• **Myocardial infarction:** The risk of MI following influenza vaccination was assessed in three (20%) SRMAs. Two were SRMAs of RCTs, whereas one included observational studies. Participants were at high-risk of CV events in all SRMAs. Findings showed a significant reduction in the risk of MI in vaccinated individuals, ranging between 29% (95% CI: 9%–44%) to 34% (95% CI: 7%–54%) with no heterogeneity (I^2^: 0%) (24,36), whereas another SRMA reported a 31% reduction in MI, although it did not reach statistical significance and had substantial heterogeneity (I^2^: 63%) (Figure 3) ((28)).

• **Stroke:** Stroke and transient ischemic heart attack (TIA) in influenza vaccinated individuals were evaluated in five (33%) SRMAs. Three of these SRMAs involved participants with high-risk for CV events ((22,32,42)), while two included adults and older adults ((41,45)). Other than one SRMA that was of RCTs only, the remaining SRMA included observational studies or both RCTs and observational studies. The overall risk reduction in stroke and TIA ranged between 13% (95% CI: 4%–21%) and 19% (95% CI: 14%–23%), and was statistically significant across four SRMAs, with substantial heterogeneity (I^2^: 53%–86%) (Figure 3).

## Discussion

This review presents a comprehensive evidence synthesis from multiple published and robust SRMAs that assessed the association between influenza vaccination and CV events. Detailed pooled effect measures were reported for four main CV outcomes: CV mortality, MACE, MI and stroke/TIA. Most SRMAs reported a significant reduction in CV events following influenza vaccination, especially in individuals with underlying CVD, or at higher risk for CV events. Indeed, the risk for CV mortality was up to 47% lower in vaccinated individuals, whereas MACE was reduced by 37% and MI events by 34% compared to unvaccinated individuals. Finally, the risk of stroke/TIA was reduced by up to 19% in vaccinated individuals.

### Interpretations

Influenza is well recognized as a trigger for CV outcomes, especially in the first two weeks following infection ((8)). The risk of CV exacerbation or complications following influenza infection is particularly high in individuals with pre-existing CVD ((47)). Thus, influenza vaccination stands out as a potentially effective intervention to reduce the burden of CV outcomes, especially among high-risk groups ((8,13)). Several mechanisms underlie the cardioprotective effects of influenza vaccination. While influenza triggers systemic inflammation, which can exacerbate atherosclerosis and CVD, the vaccine activates the immune system, enhancing overall immune health and preventing secondary infections that could worsen CV conditions. Additionally, it could help stabilize atherosclerotic plaques, thus reducing the risk of acute CV events, according to some earlier findings ((39)).

Altogether, the available evidence supports recommending annual influenza vaccination for high-risk individuals, particularly those with underlying CVD. This preventive measure can significantly reduce the risk of CV events and improve overall health outcomes in these populations.

However, despite the recommendations for individuals with chronic health conditions in Canada to be vaccinated ((14)), influenza vaccination coverage remains sub-optimal in these groups. During the 2023–2024 season, only 44.1% of adults aged 18–64 with chronic medical conditions were vaccinated against influenza, whereas the national goals for seasonal influenza in this population were to achieve 80% vaccination coverage (48).

### Implications

An effective communication of influenza vaccine-associated benefits against specific outcomes could help foster vaccination ((49)). A large trial in Denmark titled Nationwide Utilization of Danish Government Electronic Letter System for Increasing Influenza Vaccine Uptake (NUDGE-FLU) investigated the effect of digital behavioural nudges on influenza vaccine uptake among individuals aged 65 years and older, with a focus on CVD status ((50)). Over 960,000 Danish citizens were randomized to usual care or one of nine electronically delivered letters, designed using behavioural concepts, prior to the 2022–2023 seasonal influenza vaccination period. One of these letters specifically emphasized the potential CV benefits of influenza vaccination. Interestingly, this CV-focused letter had the greatest effect on increasing vaccine uptake. The effect was consistent across individuals with and without CVD, as well as across CVD subgroups. This suggests that emphasizing CV benefits may be an effective strategy to boost vaccination rates, even among those without existing CVD ((50)).

Thus, clear communication about the potential CV benefits associated with influenza vaccination could help raise awareness and motivation to vaccinate among high-risk groups, who are already targeted for the annual vaccination campaign, about the usefulness of influenza vaccines. Nevertheless, since data on CV benefits are not usually included in studies analyzing the benefits of influenza vaccination and given the recent accumulation of studies on the subject, it would be interesting to consider this type of effect in future cost-effectiveness evaluations of influenza vaccines ((13)).

## Limitations

Despite the strength of this evidence synthesis, this review possesses limitations inherent to included studies. First, the quality and heterogeneity of the included primary studies varied, which may influence the accuracy of pooled estimates. The observed heterogeneity could be attributable to differences in the study populations, CV outcomes definition, duration of follow-up and timing of vaccination. Secondly, many SRMAs included observational studies, which are prone to confounding bias. Finally, although associations were consistent, the causality of the effect cannot be ascertained, and large-scale RCTs are needed to further explore the cardioprotective effects of influenza vaccination.

## Conclusion

In conclusion, this umbrella review provides a high-quality evidence synthesis supporting the CV benefits of influenza vaccination. The significant reductions in CV mortality, MACE, and stroke highlight the importance of promoting influenza vaccination, particularly among people with underlying chronic medical conditions, such as CVD. By integrating influenza vaccination into routine clinical practice and public health strategies, CV outcomes can be improved while reducing the burden of both CVD and influenza.

## Appendix

Supplemental material is available upon request to the author: naci-ccni@phac-aspc.gc.ca

Supplemental A: Search strategy

Table S1: AMSTAR 2 detailed assessment

Table S2: Overlap between primary studies matrix
